# Tuberculous peritonitis in a cerebral palsy patient: A challenge in diagnosis and management

**DOI:** 10.1016/j.ijscr.2019.04.019

**Published:** 2019-04-16

**Authors:** Vorapatu Tangsirapat, Vichack Chakrapan Na Ayudhya, Panutchaya Kongon, Kobkool Chakrapan Na Ayudhya, Paiboon Sookpotarom, Paisarn Vejchapipat

**Affiliations:** a Department of Surgery, Panyananthaphikkhu Chonprathan Medical Center, Srinakharinwirot University, Nonthaburi, 11120, Thailand; b Department of Surgery, Faculty of Medicine, Chulalongkorn University, Bangkok, 10330, Thailand

**Keywords:** Tuberculous, Peritonitis, Cerebral palsy, Case report

## Abstract

**Introduction:**

Diagnosis of tuberculous peritonitis (TBP) in a normal person, although possible, is often difficult to make because of its non-specific symptoms and signs. However, establishing a diagnosis of TBP in a patient with cerebral palsy (CP) does not seem to be possible due to impaired mental development accompanied by communication problems.

**Presentation of case:**

A 19-year-old spastic man diagnosed with CP presented with fever and a nonverbal complaint of abdominal pain. The conditions were hard to evaluate due to his mental status. Abdominal radiography showed dilatation of both small and large bowels, and a subsequent computed tomography (CT) scan did not provide any additional information. With respect to a common suspected cause, a diagnosis of perforated appendicitis was established. However, at the theatre, there was only bowel dilatation with multiple small nodules at the serosa of small and large bowels. Postoperatively, polymerase chain reaction and culture revealed Mycobacterium tuberculosis, thereby leading to a diagnosis of TBP.

**Discussion:**

Due to spasticity caused by CP, on examination, the patient presented with board-like rigidity, from which a diagnosis of a surgical condition was established. The misdiagnosis of an acute abdomen situation had let the patient to undergo an unnecessary exploration. To our knowledge, there has not been a report of TBP in a CP patient.

**Conclusion:**

The diagnosis of TBP had been complicated by the presence of CP in the reported case. The underlying CP not only preclude the diagnosis of TBP, but also produced symptoms that mimicked a condition requiring surgery.

## Highlights

• Mental retardation caused by cerebral palsy results in difficult examination.
• Spasticity in cerebral palsy can mimic acute abdomen.
• Diagnosis of tuberculous peritonitis constitutes difficult task in cerebral palsy.

## 1 Introduction

In the nearly 2 centuries since the first discovery of tuberculous peritonitis (TBP) in 1843 [1], extrapulmonary forms of tuberculosis (TB), including TBP, are still continuously diagnosed representing 14% of new cases of TB infection [2]. Although it is possible to diagnose TBP in a normal person, it is often difficult because of the disease’s non-specific symptoms and signs, which can include abdominal pain, ascites, weight loss, fever, and presence of history of TB infection earlier [3]. In a patient with cerebral palsy (CP), a condition comprising of delay of mental development, communication problems, and spastic muscular tone, the diagnosis of TBP does not seem to be possible, and may led to a delayed diagnosis, the misdiagnosis as surgical abdomen condition, or increased morbidity and mortality [[4], [5], [6]].

This work is compliant with the SCARE checklist, and also, has been reported in line with the SCARE criteria [7].

## 2 Presentation of case

**Fig. 1:**
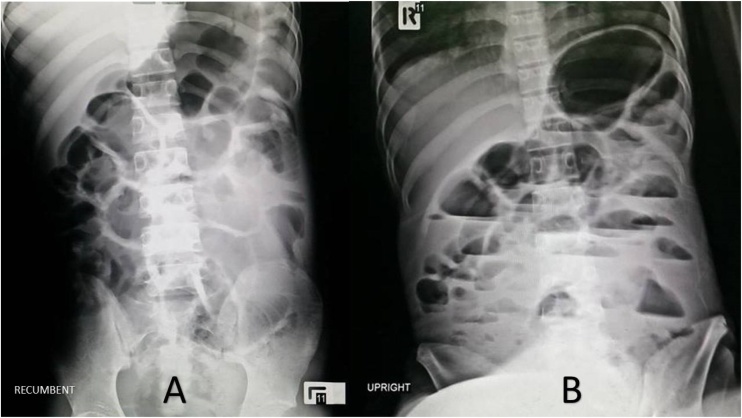
Abdominal radiography revealed dilatation of small and large intestine in recumbent (A) and upright (B) view.

**Fig. 2:**
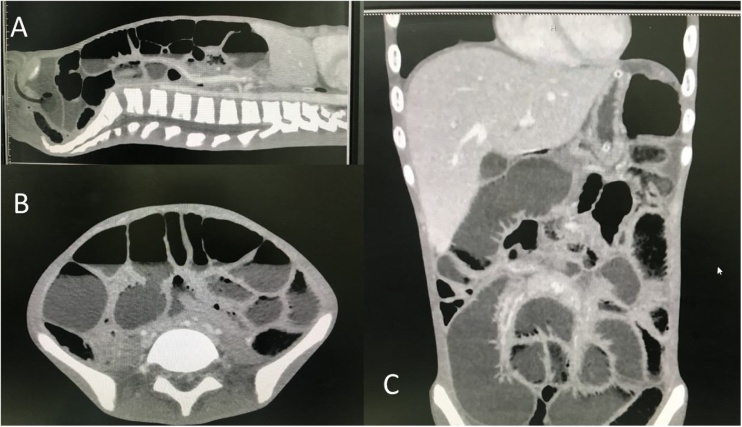
Sagittal (A), axial (B), and coronal (C) CT scan views showed the same findings as shown in radiography.

A 19 year-old-spastic man arrived at our emergency department with abdominal distension, vomiting, and fatigue for 3 days. He also had a low-grade fever for 2 weeks. Since birth, he has been diagnosed with CP and has been living in a disabled children’s center as a disabled person. His abdominal pain could not be assessed due to his mental status. Physical examination confirmed low-grade fever and showed signs of dehydration and enlarged cervical lymph nodes. There was a presence of abdominal distension with decreased bowel sound and mildly generalized tenderness. Laboratory tests revealed a total white blood cell count of 17,000 per microliter and a neutrophil count of 90%. Test for human immunodeficiency virus was negative. Abdominal radiography showed ileus with dilatation of both small and large bowel (Fig. 1). Due to suspicion of infection, computed tomography (CT) scan was obtained showing a diffusely thickened bowel wall, generalized small and large bowel dilatation, and ascites at the cul-de-sac (Fig. 2). There was no point of obstruction. The appendix could not be outlined and no pneumoperitoneum was found.

**Fig. 3:**
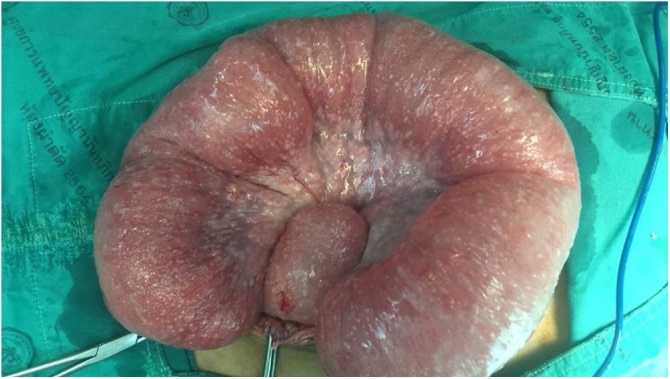
Gross findings as generalized serosal micro-nodules was consistent with TBP.

Initially, intravenous 1 g ceftriaxone and 500 mg metronidazole were started. Following a day of observation, there were sudden changes of abdominal signs. His abdominal signs got worse revealing increased distension, generalized involuntary guarding and marked tenderness at the right side of the abdomen. As a result, a diagnosis of perforated appendicitis was made, as it is commonly founded in this group of patients with peritonitis. Consequently, the patient was transferred to operating room. At the theatre, there was a presence of small and large bowel dilatation without a point of obstruction or perforation. Interestingly, there were multiple small nodules at the serosa of small and large intestine, mesentery, parietal peritoneum, and cul-de-sac (Fig. 3). Clear yellowish ascites about 500 mL at cul-de-sac were obtained. Only the peritoneal tissue biopsy was performed. No acid-fast organism was seen in ascites. The pathologic report unfolded caseous granuloma with a positive 1+ Ziehl–Neelsen staining and a positive PCR for tuberculous complex. The tissue culture result was positive for Mycobacterium TB. Therefore, the sputum was sent for Ziehl–Neelsen stain and found positive 1+. Post-operatively, abdominal distension and bowel movement had improved after taking anti-TB medication. Unfortunately, however, the patient passed away on post-operative day 11 due to *Acinetobacter baumannii* hospital acquired pneumonia.

## 3 Discussion

The abdominal cavity constitutes an uncommon site of TB infection, when considering all extrapulmonary sites that could be affected by TB. Intraabdominal TB is found in around 10–30% of patients with pulmonary TB but only 3% of extrapulmonary TB cases [8]. It may be possible that TB spreads to the abdominal cavity via the hematogenous route from the pulmonary site, passes through ingestion of infected sputum through the Peyer's patch and mesenteric lymph nodes, or directly spreads from infected adjacent lymph nodes and fallopian tubes [3]. As the patient lives and grows up in a disabled children’s center, a crowded community, TB can spread via airborne transmission comparatively easily.

Thailand is one of 20 countries with the highest number of incidents of TB. In 2017, the number of TB occurrences in Thailand was 156 cases per 100,000 people [2]. Although there is a presence of an earlier treatment for pulmonary TB, the diagnosis of TBP is still not uncomplicated even in a normal population. The clinical symptoms and signs of TBP are non-specific, including abdominal pain or tenderness, ascites, weight loss, fever, diarrhea, constipation, hepato-splenomegaly, etc., and thus further investigations are usually required to make the diagnosis. There is only a 14% chance that an active TB can be found on chest radiography, and rarely military TB is seen [9]. Ultrasonography can demonstrate multiple mobile septations in peritoneum, thickened mesentery, and mesenteric lymphadenopathy. CT scans may also help in detecting ascites, omental thickening, mesenteric nodes, and peritoneal micronodules [[10], [11], [12]].

The patient subsequently developed peritonitis with rigid abdomen, which may have been caused by spasticity from CP, so emergency surgical condition was considered. These abdominal signs, with muscle guarding, have been reportedly associated with a perforated appendicitis in young patients [13,14]. Ohmann score including age of patient less than 50 years old, steady pain, leukocytosis, and abdominal rigidity can help determine the likelihood of appendicitis [15,16]. Patients with CP have a communication problem, a situation often encountered when treating children. The generalized small bowel dilatation in our patient might be secondary to diffuse inflammation, which was frequently found in children with perforated appendicitis [17]. Even though the CT scan of the appendicitis has high sensitivity of 97% and specificity of 93% [18], it was not helpful in this patient since the appendix could not be outlined. As a result, with sudden change of rigid abdomen with muscle guarding, and generalized bowel dilatation, the patient was therefore suspected to have perforated appendicitis.

The average survival rate of CP patients is currently high, 98.2% of children aged 4–14 survived 20 years in mild cases [19], and hence nowadays more CP adults can be encountered in the community. However, patients with CP often experience a delayed diagnosis due to nonspecific complaints and inability to clearly communicate their symptoms. This leads to increase morbidity and mortality in CP patients with acute abdomen conditions [[4], [5], [6]]. Although most patients with intraabdominal TB well respond to standard anti-TB medication [3], a situation of scarce information as described and the misdiagnosis of an acute abdomen condition had let the patient undergo an unnecessary exploration.

As previously mentioned, the radiography and CT scan performed in our patient provided little information. Unfortunately, CP, an underlying movement and mental disorder, also complicated the situation by precluding an interactive communication. In addition, as a disabled patient reared and cared in the children’s center by caregivers who were not his own parents, information taken for diagnosis was only obtained from examination and investigation. As a result, the failure to collect a complete history of the present illness and findings inevitably entails difficulty in the diagnosis of TBP.

Since people with CP living in a rehabilitation center were mostly malnourished, they were at risk of developing more complications [20]. Likewise, there was a malnutrition presented during this admission. Post-operatively, this condition may be a supplemental factor in explaining why the patient developed pneumonia and finally died of sepsis.

## 4 Conclusion

As we have shown, the diagnosis of TBP had been complicated by the presence of CP in the reported case. Not only did the underlying CP preclude diagnosis of TBP, but some characteristics produced by CP also mimicked a condition requiring surgery—namely, had the TBP been kept in mind and initially diagnosed, the patient would have been treated with an anti-TB regimen, and not have undergone unnecessary exploratory surgery.

## Conflicts of interest

The authors declare that there is no conflict of interest regarding the publication of this article.

## Sources of funding

This work received no funding.

## Ethical approval

The consent form and information sheet using in the process of obtaining a consent were approved by IRB at our institution.

## Consent

A Dean of the children’s center where the patient lived has been informed prior to the conduction of this manuscript and informed consent has also been obtained. A copy of the written consent is available for review by the editor-in-chief of the journal on request.

## Author’s contribution

Vorapatu Tangsirapat and Panutchaya Kongon collected data and wrote manuscript.

Vichack Chakrapan Na Ayudhya and Kobkool Chakrapan Na Ayudhya contributed to conceptualization.

Paiboon Sookpotarom contributed to conceptualization, data curation, supervision and editing of the manuscript.

Paisarn Vejchapipat finally edited this manuscript.

## Registration of research studies

Research Registry Unique Identifying Number: researchregistry4769.

## Guarantor

Paiboon Sookpotarom.

## Provenance and peer review

Not commissioned, externally peer-reviewed.
